# Comparison of the optical quality vision between real post-LASIK myopic laser surgery and the simulated implantation of a phakic IOL in low myopia

**DOI:** 10.1038/s41598-022-23662-3

**Published:** 2022-11-07

**Authors:** Celia García, Vicente J. Camps, María T. Caballero, David P. Piñero, Pedro Tañá, Cristina Tello, Juan J. Miret

**Affiliations:** 1 Grupo de Óptica y Percepción Visual (GOPV). Department of Optics, Pharmacology and Anatomy, University of Alicante, Crta San Vicente del Raspeig S/N, San Vicente del Raspeig, 03690 Alicante, Spain; 2 Oftalvist Alicante, Avinguda de Dénia, 103, 03015 Alicante, Spain

**Keywords:** Imaging and sensing, Optical tweezers, Quality of life

## Abstract

A phakic intraocular lens (PIOL) of − 4.5 D was characterized from its wavefront aberration profile. A preclinical study was conducted using pre- and post-surgery data from four patients that had undergone myopic laser refractive surgery. All these patients would have needed a PIOL of − 4.5 D. Pre-surgery data were used to simulate the effect of a PIOL implantation. Post myopic refractive surgery data were used to calculate the post-LASIK eye model. Modulation transfer function (MTF), point spread function (PSF) and simulation of optotypes vision were obtained and compared. The PIOL did not worsen the optical quality of the eyes evaluated. High order Aberrations were always higher in the post-LASIK eye model. Optics quality trended to be better in PIOL implantation than post-LASIK surgery as pupil size increased.

## Introduction

Besides the classical solution of spectacle prescription for the correction of refractive errors, surgical techniques such as corneal laser refractive surgery or the implantation of a phakic intraocular lens (PIOL) are also safe and efficacious options for such purpose. Several studies have compared in last years the clinical outcomes obtained with these two surgical procedures. Both, clinical and theoretical studies, have been conducted to define the advantages and disadvantages of these two refractive surgery techniques. Most of clinical studies conclude that PIOL implantation provides better predictability and stability of refractive correction and better visual acuity (VA) compared to eyes undergoing laser in situ keratomileusis (LASIK)^1–5^. The main reason of these differences among techniques is that the corneal shaping induced by the laser ablation increases the level of higher-order aberrations (HOAs), especially spherical aberration^2,6–8^. Pérez-Vives et al. were the first authors to propose a method to compare the visual performance provided by LASIK and a specific modality of PIOL (Implantable Collamer Lens, ICL) for myopia correction on the same eye using an adaptative optics method^9^. Specifically, they simulated the visual performance of ten healthy subjects considering the implantation of the Visian ICL PIOL and the performance of myopic laser refractive surgery. For this purpose, the wavefront aberration pattern (wavelength 850 nm) of two ICLs (− 3 and − 6 D), the subject eye’s wavefront and the wavefront pattern of the LASIK patients obtained by the same authors in previous studies were considered. In that study, they concluded that the ICL provided better outcomes than corneal laser refractive surgery, especially for large refractive errors and pupil sizes.

Our group proposed in 2017^10^ a new method based on a special optical bench with a Shack-Hartmann wavefront sensor (SH) for the measurement of the aberrometric profile of intraocular lenses (IOLs). This new method uses green light of 532 nm (and not infrared) for the measurements that overcomes the possible bias introduced by infrared measurements as it happens in diffractive IOLs^11^. The combination of these in-vitro IOL wavefront aberration measurements, real corneal topographic data and ray-tracing simulation has been used to simulate the optical performance of IOLs implanted in eyes with previous myopic or hyperopic laser corneal refractive surgery^12^. The simulated through-focus modulation transfer function (TMTF) for three presbyopia-correcting IOLs (Mini-Well, Tecnis Symfony and Lentis Mplus) implanted in one eye with previous myopic LASIK and another one with hyperopic LASIK were obtained and compared. Recently, this methodology has been used to analyze if the proper use of a segmented intraocular lens (IOL) could improve the visual outcomes compared to the implantation of a spherical monofocal IOL^13^. As previously mentioned, the efficacy and safety of phakic IOLs implantation compared to laser surgery in high myopia has been extensively analyzed and studied. However, the use of phakic IOLs implantation in low or moderate myopia is nowadays an important point of controversy since the possible advantages or disadvantages of lens implantation versus the laser refractive surgery are not so clear. In the current study, simulations based on ray-tracing and real patient data were developed to compare the optical performance of low/moderate myopia correction by means of laser corneal refractive surgery and implantation of a PIOL.

## Methods

### Phakic IOL used for the simulations

The EVO Visian ICL, formerly Visian ICL CentraFLOWTM V4c (STAAR Surgical, Nidau, Switzerland), was used in our simulations, which is a plate-haptic single-piece PIOL that incorporates a small central hole promoting a natural circulation of aqueous humour and avoiding the need for peripherical iridotomies (PIs). It is made of a hydrophilic and biocompatible material called Collamer® which is composed of a collagen and a co-polymer. The spherical myopic ICLs are available in 0.25 D increments from − 0.5 D to − 3.0 D and in 0.5 D increments from − 3.0 D to − 18.0 D, with an optical zone that can shift from 4.9 to 5.8 mm. In addition, hyperopic and toric PIOLs are also available. In this study, we used and characterized an ICL of − 4.5 D IOL of 5 mm of optic zone and 13.2 mm of overall diameter.

### Measurement simulation set-up

As commented above, the methodology used in this study was based on that described in a previous study in which the optical performance of three presbyopia-correcting IOLs implanted in eyes with previous laser refractive surgery was simulated and to show that the use of a standard toric segmented IOL with a proper orientation and selection of the addition can improve the optical quality of the keratoconus eye compared to the use of a monofocal spherical IOL.^12,13^. Following the same procedure of this previous work, an ICL of − 4.5 D was characterized in terms of wavefront aberration profile using a Hartmann-Shack sensor and based on the guidelines of the ISO 11979-9^14,15^. A green light of 532 was used. The PIOL was placed in the bottom of a wet cell which was a chamber that had transparent optical windows and that was filled with saline solution (0.9% saline solution). The PIOL was aligned with the optical axis of the wavefront sensor using an XYZ translational stage^10^.

For the preclinical validation, real pre- and post-surgery corneal data of four patients that have undergone myopic laser refractive surgery was collected from Oftalvist Clinic in Alicante (Spain). All topographic examinations were performed using a Scheimpflug-based imaging system (Pentacam, Oculus Optikgerate GmbH, Wetzlar, Germany). These four patients were selected considering that they were suitable for the correction of their myopia with corneal laser refractive surgery and PIOL. For each eye a customized model of eye was implemented. All eye models were calculated using the pre-surgery biometric data (corneal thickness, anterior chamber depth and the axial length), the topographies of the first and second surface of the cornea, and a theoretical aspherical lens based on Navarro’s model^16^. An ideal paraxial ophthalmic lens with the pre-surgery refraction and located 14 mm in front of cornea was added. This simulated eye was named “eye model” and it was used to customize the theoretical lens model of each eye incorporating astigmatic and aspherical surfaces. Starting from this eye model, and maintaining the lens calculated, the ophthalmic lens was removed and the ICL PIOL added. This second model eye was called "ICL model". The effective ICL position was calculated to get the emmetropia considering a vault between 300 µm and 500 µm. Finally, based on the “eye model”, a third simulated eye was built. In this model the ophthalmic lens was removed, and the pre-surgery corneal data (topographies and thickness) were replaced by the post-surgery corneal data, and it was called "post-LASIK model”. The topographies were uploaded once exported in csv format from the Pentacam system (OCULUS Optikgerate GmbH, Wetzlar, Germany). Each corneal wavefront was propagated to the PIOL, which was introduced as a phase element. The optical performance of such combination was obtained after combining ray tracing and Fourier optics (Zemax, LLC Washington, USA and MATLAB, The MathWorks, Natick, MA) ^12–15^.

For the comparison of the three eye simulations (myopic laser refractive surgery, ICL implantation and ophthalmic lens), the Modulation Transfer Function (MTF) up to 100 cycles/mm and the Point Spread Function (PSF) were calculated and represented. Furthermore, the theoretical appearance of the optotypes were simulated to estimate the final VA with each eye model and to compare them. Figures 5 and 6 show the simulated Snellen optotypes chart used in our study and their corresponding visual acuities. The simulation of the VA with the optotypes was not considering the neural processing and therefore the real visual perception of the optotype chart is expected to be better than the image of the optotypes obtained on the retina. Following the ISO 11979-9 recommendations about the evaluation of the imaging quality of premium IOLs, all measurements were performed for two exit pupil sizes under photopic (3 mm) and scotopic (4.5 mm) light conditions.

### Clinical data

Data from four eyes of three patients between 24 and 33 years were used in the current study. Inclusion criteria were patients from 20 to 40 years with a stable myopia around − 4 D and a cylinder lower than 0.75 D that had undergone myopic LASIK. Patients with irregular cornea, any previous ocular surgery, glaucoma, retinal diseases, dry eye or amblyopia were excluded. In addition, only patients with an anterior chamber depth (ACD) larger than 2.8 mm were included.

All eyes included in the study were successfully operated on using the Femto-LASIK technique, (IFS Advanced Femtosecond Laser, Johnson & Johnson Vision, California, USA) creating a 110 µm thickness flap with superior hinge of 9.0 mm of diameter and posterior treatment using the Triple-A ablation profile with the excimer Laser MEL 90 (Carl Zeiss Meditec, Jena, Germany) at the Department of Ophthalmology (Oftalvist) of HLA Vistahermosa (Alicante, Spain). There were not intra-surgical complications or any corrections or flap re-adjustment. Post-operative examinations were realized at 1 day, 1 month, 4 months and 1 year after surgery. Different topical treatments were prescribed to each patient after surgery. As seen in table 2, all patients achieved UCVA of 1 after surgery.

Tables 1 and 2 show the mean ocular and biometric parameters of the four eyes enrolled in this study.

**Table 1:** Preoperative ocular and biometric parameters of the four eyes*.

	Sph (D)	Cyl (D)	Axis(º)	CDVA	ICL power (D)	K1 (D)	K1 (º)	K2 (D)	K2 (º)	AL (mm)	ACD (mm)	CT (mm)	ϕ_real_ (mm)
Eye1	− 3.75	− 0.25	85	1	− 4.5	41.93	16	42.17	106	25.19	3.46	0.577	2.59
Eye2	− 4	0	0	1	− 4.5	42.24	158	42.46	68	25.12	3.55	0.580	2.58
Eye3	− 3.25	− 0.25	15	1	− 4.5	43.51	179	44.39	89	25.15	3.82	0.570	3.43
Eye4	− 3.5	− 0.25	135	1	− 4.5	45.4	156	46.47	66	23.88	3.68	0.508	3.11

*Sph (sphere), *Cyl* (Cylinder), *Axis* (cylinder axis), *CDVA *(corrected distance VA), K1 and K2 (keratometries), K1 (º) and K2(º) (keratometric axis), *AL* (axial length), *ACD* (anterior chamber depth), *CT *(corneal thickness) and ϕ_real_ (real pupil size).

**Table 2:** Postoperative ocular parameters of the four eyes*.

	Post-Sph (D)	Post-Cyl (D)	Post-Axis(º)	Post-UDVA	Post-CDVA	K1 (D)	K1(º)	K2 (D)	K2(º)
Eye1	0,25	0	0	1	1	39,04	180	39,13	90
Eye2	0,25	0	0	1	1	38,91	122	39,12	32
Eye3	− 0,25	0	0	1	1	41,16	14	41,6	104
Eye4	0	0	0	1	1	42,73	153	43,52	63

*Sph (sphere), *Cyl* (Cylinder), *Axis* (cylinder axis), *CDVA* (corrected distance VA), K1 and K2 (keratometries), K1 (º) and K2(º) (keratometric axis).

## Results

### ICL Zernike Coefficients

Zernike coefficients up to 8th order were obtained for the ICL of − 4.5 D. As shown in Table 3 (only spherical-like and coma-like coefficients are indicated), the PIOL is practically free of aberrations and only spherical components are present independently the pupil size (the rest of coefficients not shown are all 0). High order aberration root mean square (HORMS) remained constant around 0.025 µm independently from the pupil size.

**Table 3:** ICL Coma-like and Spherical-like Zernike coefficients obtained for the pupil sizes of 3 mm and 4.5 mm. High Order RMS aberrations (HORMS) were calculated onsidering the Zernike coefficients from the third to eighth orders.﻿

C(n,m) (µm)	3 mm	± SD	4.5 mm	± SD
C(3, − 1)	0.000	0.019	0.000	0.016
C(3,1)	0.000	0.002	0.000	0.004
C(4,0)	0.012	0.003	0.004	0.013
C(5, − 1)	0.000	0.016	0.000	0.005
C(5,1)	0.000	0.007	0.000	0.005
C(6,0)	0.005	0.002	0.007	0.006
C(7, − 1)	0.000	0.003	0.000	0.016
C(7,1)	0.000	0.006	0.000	0.014
C(8,0)	0.012	0.004	0.010	0.004
HORMS	**0.029**	**0.007**	**0.024**	**0.007**

### Zernike coefficients of three simulated eyes

As explained above, real preoperative topography and ocular parameters were used to obtain the so-called eye model (with ophthalmic lens) and ICL model, whereas the so-called post-LASIK model was built using real post-LASIK topography and ocular parameters.

Table 4 shows the Zernike coefficients of four different eyes (not only the cornea aberrations). As seen, typical increase of aberrations as pupil size increase is observed. This behavior is obtained in all eyes and in all the cases analyzed, being always RMS higher for 4.5 mm pupil size than 3 mm pupil size. If only the eye model was considered, the eyes 1, 2 and 3 showed similar HORMS for pupil sizes of 3 mm (0.02 µm) and 4.5 mm (between 0.07 and 0.085 µm). However, eye 4 showed higher values of HORMS (0,051 µm for 3 mm and 0.147 µm for 4.5 mm). When the ICL implantation and the post-LASIK surgery models were considered, the aberrations increased for all pupil sizes, but this increment was clearly higher in the post-LASIK eye model. Only for the eye 4, there were not differences between the three eye simulations. This absence of differences in eye 4 was related to the higher level of aberrations compared to the other eyes already present preoperatively. It should be noted here that the eye model and the ICL model used the same preoperative data.

**Table 4:** Spherical-like and coma- like Zernike coefficients of the four eyes for the eye model as well as for the ICL implantation and the post-LASIK surgery simulations. High Order RMS aberrations (HORMS) were calculated onsidering the Zernike coefficients from the third to eighth orders.

Eye 1	Eye model		ICL		post-LASIK	
C(n,m)	3 mm	4.5 mm	3 mm	4.5 mm	3 mm	4.5 mm
C(3, − 1)	− 0.001	− 0.050	− 0.018	− 0.039	− 0.038	− 0.118
C(3,1)	− 0.018	− 0.033	− 0.016	− 0.058	0.030	0.020
C(4,0)	0.000	− 0.005	− 0.013	− 0.056	− 0.023	− 0.044
C(5, − 1)	− 0.003	− 0.016	− 0.002	− 0.010	0.000	0.006
C(5,1)	0.002	0.010	0.001	0.009	− 0.004	− 0.028
C(6,0)	0.000	− 0.003	0.005	− 0.006	0.002	0.020
C(7, − 1)	0.000	0.002	0.000	− 0.001	0.000	0.001
C(7,1)	0.000	− 0.001	0.000	0.001	0.000	0.001
C(8,0)	0.000	0.000	0.000	0.001	0.000	0.000
HORMS	**0.020**	**0.073**	**0.035**	**0.103**	**0.056**	**0.146**

Eye 2	Eye model		ICL		post-LASIK	
C(n,m)	3 mm	4.5 mm	3 mm	4.5 mm	3 mm	4.5 mm
C(3, − 1)	− 0.001	− 0.048	0.021	− 0.036	− 0.052	− 0.152
C(3,1)	− 0.018	− 0.033	− 0.025	− 0.032	− 0.039	− 0.025
C(4,0)	0.000	− 0.002	0.013	− 0.008	− 0.018	− 0.031
C(5, − 1)	− 0.003	− 0.015	− 0.008	− 0.013	0.001	0.009
C(5,1)	0.001	0.009	0.001	0.008	0.006	0.038
C(6,0)	0.000	− 0.002	− 0.002	− 0.010	0.002	0.016
C(7, − 1)	0.000	0.002	0.000	0.001	0.000	0.000
C(7,1)	0.000	− 0.001	0.000	0.000	0.000	− 0.002
C(8,0)	0.000	0.000	0.000	0.000	0.000	0.000
HORMS	**0.020**	**0.071**	**0.038**	**0.061**	**0.075**	**0.176**

Eye 3	Eye model		ICL		post-LASIK	
C(n,m)	3 mm	4.5 mm	3 mm	4.5 mm	3 mm	4.5 mm
C(3, − 1)	0.002	− 0.011	− 0.015	− 0.009	− 0.010	− 0.045
C(3,1)	− 0.015	− 0.059	− 0.012	− 0.067	0.033	0.072
C(4,0)	0.001	− 0.036	− 0.004	− 0.029	− 0.018	− 0.058
C(5, − 1)	− 0.001	− 0.006	− 0.001	− 0.005	0.000	− 0.002
C(5,1)	0.000	− 0.003	0.000	− 0.002	− 0.002	− 0.014
C(6,0)	− 0.001	− 0.014	0.004	− 0.012	0.001	0.011
C(7, − 1)	0.000	0.001	0.000	0.000	0.000	0.000
C(7,1)	0.000	0.000	0.000	0.000	0.000	0.001
C(8,0)	0.000	− 0.001	0.000	0.000	0.000	0.000
HORMS	**0.021**	**0.085**	**0.032**	**0.082**	**0.041**	**0.122**

Eye 4 (RE)	Eye model		ICL		post-LASIK	
C(n,m)	3 mm	4.5 mm	3 mm	4.5 mm	3 mm	4.5 mm
C(3, − 1)	− 0.018	− 0.067	0.015	− 0.007	0.014	− 0.009
C(3,1)	0.040	0.083	− 0.036	− 0.104	− 0.037	− 0.107
C(4,0)	0.022	0.078	− 0.014	− 0.001	− 0.010	0.003
C(5, − 1)	− 0.003	− 0.010	− 0.005	− 0.019	− 0.005	− 0.019
C(5,1)	− 0.002	− 0.006	− 0.002	− 0.008	− 0.002	− 0.008
C(6,0)	0.001	0.003	0.003	0.016	0.003	0.013
C(7, − 1)	0.000	0.001	0.000	0.001	0.000	0.001
C(7,1)	0.000	0.000	0.000	0.001	0.000	0.001
C(8,0)	0.000	0.000	0.000	0.000	0.000	0.000
HORMS	**0.051**	**0.147**	**0.048**	**0.119**	**0.048**	**0.122**

### MTF’s

**Figure 1:**
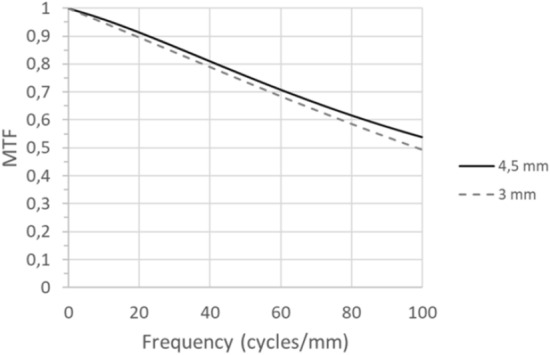
Modulation Transfer Function (MTF) up to 100 cycles/mm of the ICL for the pupil sizes of 3 mm and 4.5 mm.

Figure 1 shows the MTF of the EVO Visian ICL for pupil sizes of 3 and 4.5 mm. As seen, the optical quality was very good with values higher than 0.5 of the MTF. In fact, the optical quality was better for 4.5 mm, being the minimal value 0.55 for the frequency of 100 cycles/mm.

**Figure 2:**
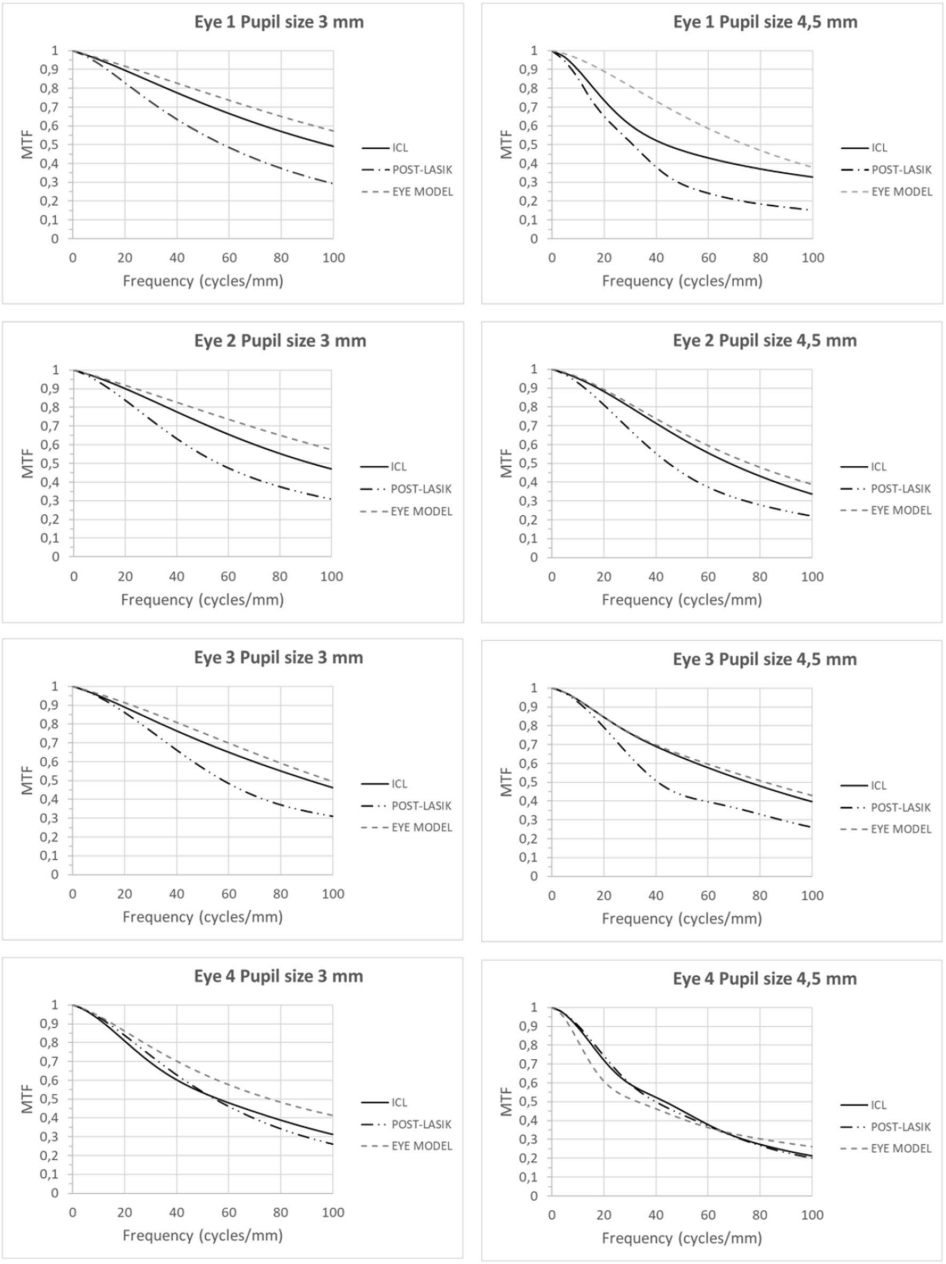
Modulation Transfer Function (MTF) up to 100 cycles/mm of the three eye models (eye model, ICL model and post-LASIK model) for the pupil sizes of 3 mm and 4.5 mm.

Figure 2 shows the MTF curves for each eye simulation for pupil sizes of 3 and 4.5 mm. As seen in all graphs, when the eye model was used, the MTF was lower than the corresponding to the ICL when was considered alone (see Fig. 1). Therefore, the ICL was not worsening the optical quality of the optical system resulting from the combination of the real preoperative cornea of the patient and the ophthalmic lens. For eyes 1, 2 and 3, the MTF associated to the ICL implantation was always higher than that for the post-LASIK eye model, with increases on average from 11 and 15%. Furthermore, the differences between the MTFs corresponding to the ICL implantation and the eye model were minimal (average difference between 1 and 5%), except for the eye 1 and pupil size of 4.5 mm that showed mean differences of 13.6%. These results indicate that the ICL option leads to better optical quality than LASIK surgery for these patients. Specifically, high differences between ICL implantation and post-LASIK surgery were obtained for spatial frequencies between 50 c/mm and 100 c/mm (from 15 to 21%), which would correspond to visual acuities of approximately 0.5 and 1.

The results for the eye 4 were quite different because not significant differences between the MTFs corresponding to the ICL implantation and post-LASIK surgery were found. In addition, the differences between the eye model MTF and the rest of eye models decreased, especially for the pupil size of 4.5 mm, not been possible to know which MTF would be better.

The reason for these differences in optical performance between eyes lied in the level of high order aberrations. According to Table 4, there was a decrease in the MTF curve when the level of higher-order aberrations increased, with the MTF curves of post-LASIK eyes decaying the fastest as they had the highest aberrations. In addition, as seen in Fig. 2, these post-LASIK curves for all eyes and one pupil size shown the same behavior indicating a very similar final optical quality result. Likewise, the MTFs curves of the eye model for eyes 1, 2 and 3 were practically identical while for the eye 4 (which had higher HORMS) it was lower.

**Figure 3:**
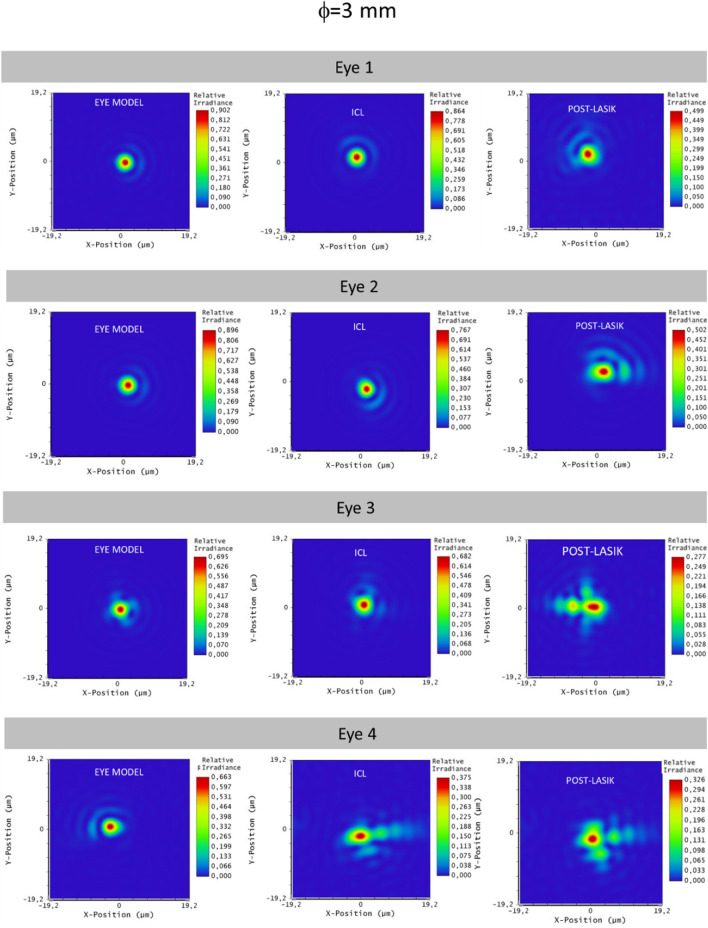
Point Spread Function (PSF) of the three eye models (eye model, ICL model and post-LASIK model) for the pupil size of 3 mm.

**Figure 4:**
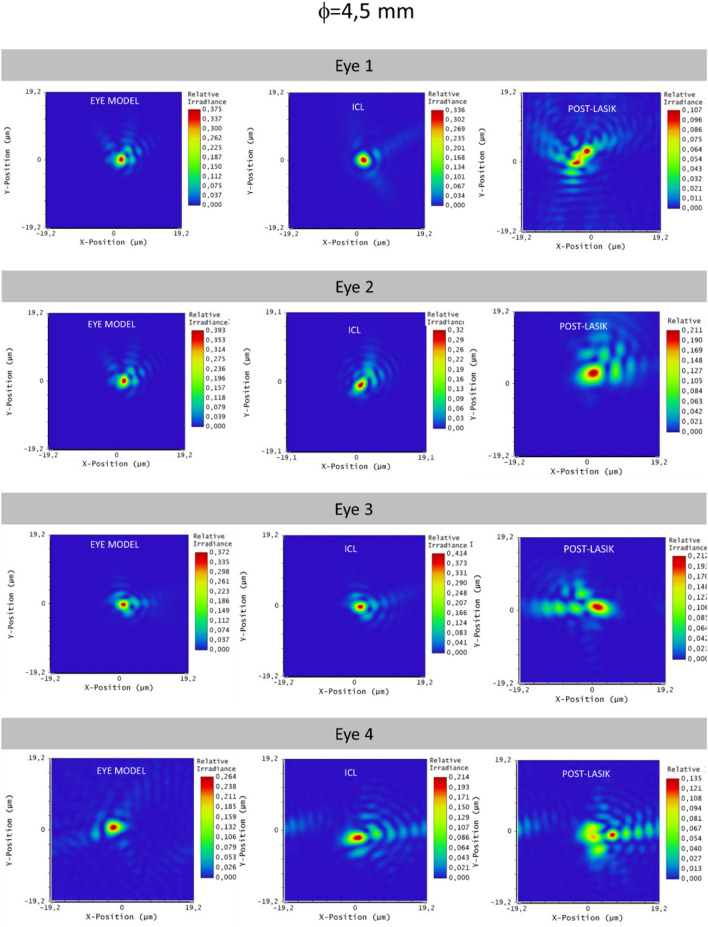
Point Spread Function (PSF) of the three eye models (eye model, ICL model and post-LASIK model) for the pupil size of 4.5 mm.

**Figure 5:**
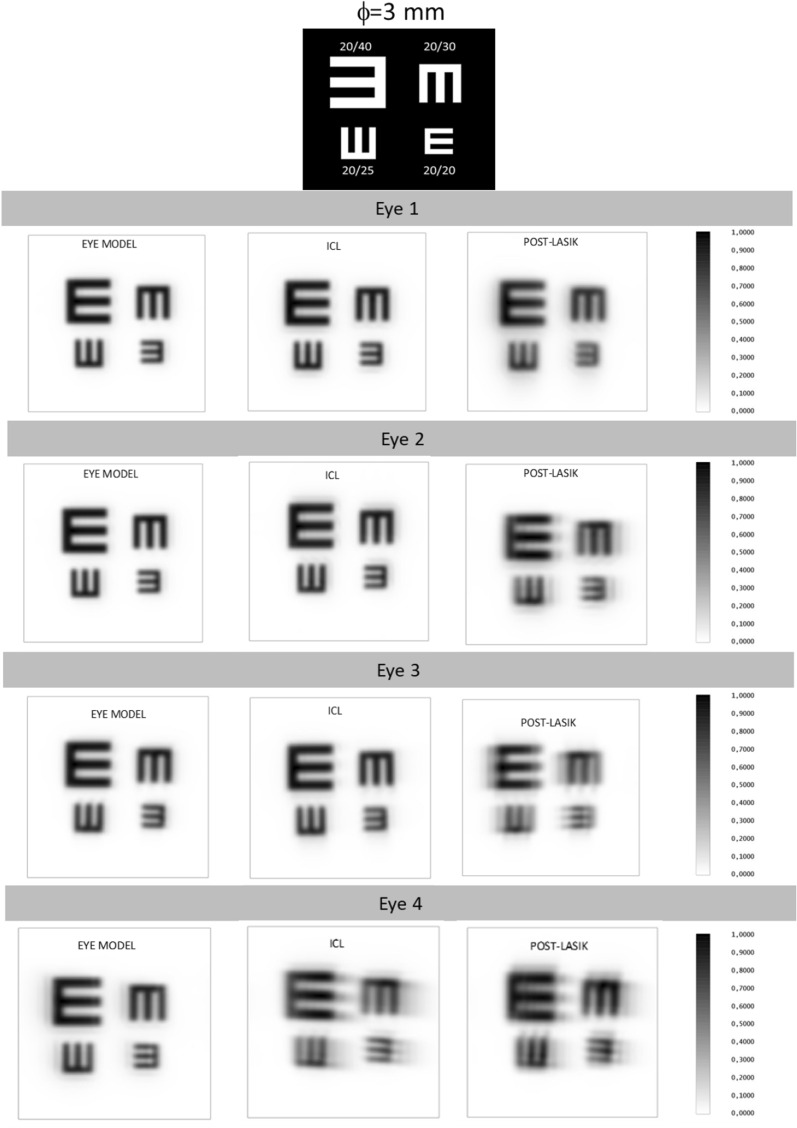
Simulated vision of the optotypes of the three eye models (eye model, ICL model and post-LASIK model) for the pupil size of 3 mm.

**Figure 6:**
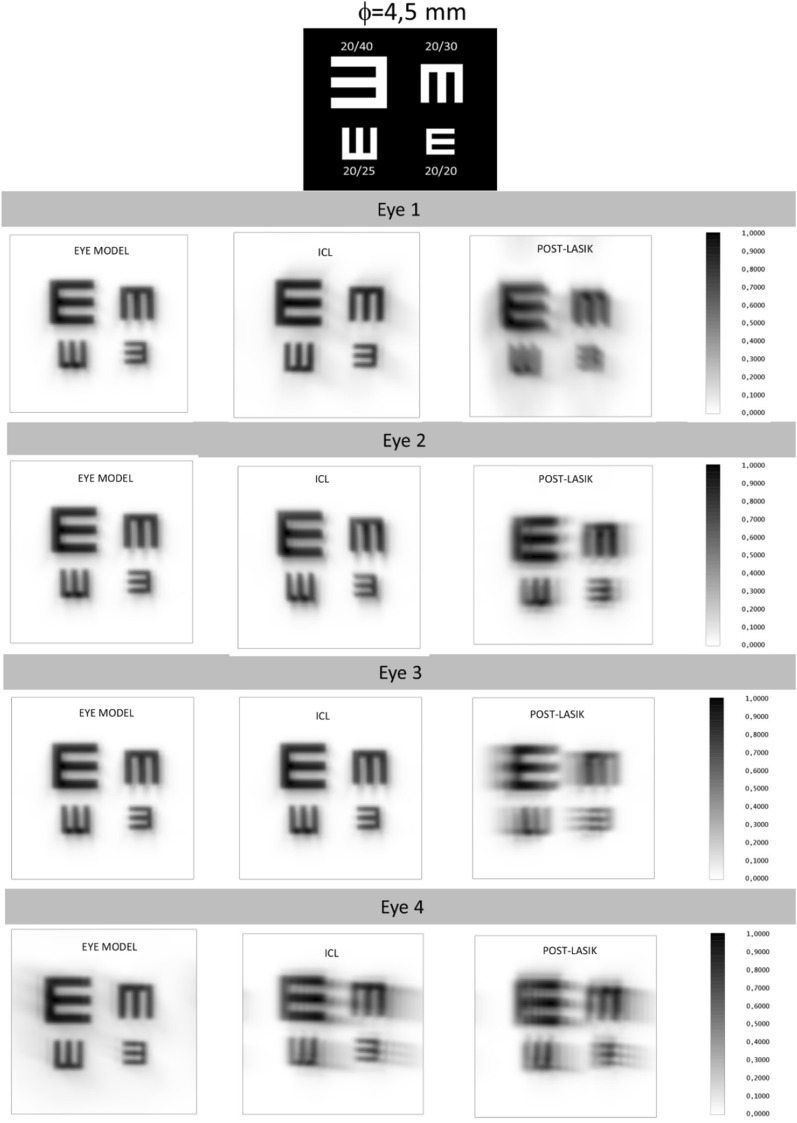
Simulated vision of the optotypes of the three eye models (eye model, ICL model and post-LASIK model) for the pupil size of 4.5 mm.

All the results are also corroborated with the simulated images displayed in Figs. 3, 4, 5 and 6. Figures 3 and 4 are representing the PSF for each eye simulation and Figs. 5 and 6 the corresponding simulated vision of optotypes. Considering the eye and ICL models, the differences between the PSF figures and the simulated vision of optotypes were minimal for eyes 1, 2 and 3 independently from the pupil size. Indeed, all these patients would achieve VA of 0,00 logMAR (see Figs. 5 and 6). However, the PSF function and vision of optotypes became worse in the post-myopic LASIK surgery model as the PSF acquired a less punctual shape and the relative irradiance decreased. In eye 4, PSFs for ICL implantation and LASIK surgery were quite similar, but worse than that for the eye model. In any case, the PSF seemed to be slightly better for the ICL simulation than for the post-LASIK simulation as it was less distorted, and the relative irradiance was always higher. In addition, the simulated vision of the optotypes was slightly better (see Figs. 5 and 6). Another result observed is that PSF and simulated optotypes got worse as the pupil size increased and differences between PIOL implant and post-LASIK surgery were more evident.

## Discussion

As previously mentioned, only one study has analyzed to this date the differences in optical quality provided by myopic correction with ICL and LASIK surgery^9^. However, our study introduces important differences that could complement this initial study. This is first time that aberrations up to 8th order of the ICL are calculated and reported (see Table 3), demonstrating that this phakic IOL should not increase aberrations in patients with low myopia. Some clinical reports support this conclusion. For example, Perez-Vives et al. obtained in high myopic patients that the magnitude of spherical aberration was directly correlated with the amount of refractive power of ICL. In addition, according to this study, for 3-mm pupil size, no statistically significant differences were observed between ICLs for any HOAs and for 4.5-mm pupil size, spherical aberration increased significantly. Similar results were obtained by Hashemian et al.^17^, who demonstrated that implantation of ICLs in high myopia patients induced negative spherical aberration, and the effect of these lenses on the other HOA as comma was negligible. The same authors indicated that an increase of comma cannot be expected in patients under ICL operation if IOL is correctly centered.

Another novelty of the current study was the use as a reference of the eye model, allowing to simulate the spectacle vision of the patient against ICL implantation or myopic LASIK surgery. To the best of our knowledge, only one paper has been published comparing MTF and PSF functions of ICL implantation and myopic LASIK surgery simulations for the same patient. In this work, Perez-Vives et al. applying adaptative optics visual simulator to obtain the visual acuity (VA), the contrast sensitivity (CS) for 3- and 5-mm pupils^9^. However, they used for all eyes the same average wavefront aberrations of standard LASIK meanwhile in present study real pre and post-LASIK surgery corneas were used. In addition, MTF and PSF were only calculated for a 5-mm pupil. They stablished that in all cases optical and visual quality was better with the ICL procedure. VA was significant better after ICL implantation and only differences in CS for 5 mm pupil and − 6 D were found.

Although a power of − 4.5 D was used in the present study, our results confirmed and extended those obtained by Pérez-Vives. The MTF of ICL in three of the four eyes (eyes 1, 2 and 3) was always higher than post-LASIK MTF independently the pupil size. These results agree with Perez-Vives et al. ^9^ obtained for − 6 D and 5 mm pupil size (the other powers and pupil sizes combinations were not showed in their paper). They showed a MTF of 0.3 for 60 cycles/degree and pupil size of 5 mm, whereas a value of 0.48 for 60 cycles/degree and pupil size of 4.5 mm was found in the current study (it should be noted that a conversion from cycles/mm to cycles/degree was done). This result is consistent because for higher pupil sizes a worsening of the MTF is expected. In addition, our results were comparable with those obtained by Uozato et al. who investigated the relationship between pupil size and the MTF of the ICL for different powers^18^. Regarding the eye 4, as seen in Fig. 2, the differences between the ICL and post-LASIK were lower. This eye shown the highest pre-surgery corneal aberrations and therefore the corresponding eye and ICL model MTFs decayed faster than in the eyes 1, 2 and 3 approaching to the post-LASIK MTF. These results highlight the importance of pre-surgery corneal aberrations in ICL implantation. As commented in introduction, previous clinical results sentenced that the ICL implantation was safer and more effective than LASIK in moderate and high myopia ^1–3^. From this study and looking at simulation of the optotypes (Figs. 5 and 6), our results suggested that this trend should remain for low myopia.

Some surgery factors of ICL implantation should be analyzed in order to stablish their importance. Woong Kim et al. analyzed the effect of incision size and power of ICL in increase of HOA^19^. They concluded that change of trefoil can be explained by the effect of the corneal incision and the negative spherical aberration by the ICL power. In addition, they found that change in the RMS of total HOA was insignificant in the small-incision group, meanwhile, the large-incision group showed a significant increase of total HOA due to a trefoil induction. Shin et al. in a prospective case series with myopic eyes from − 6.00 to − 9.00 diopters found that HORMS increased up to 0.05 µm after ICL implantation due to vertical trefoil, secondary coma and spherical aberration^8^. However, the authors concluded that the trefoil variation was due to the modification of the cornea but not to the optics of the ICL. Jiang et al. described, there was not a significant variation of corneal astigmatism, (from 1.28 ± 0.38 D preop to 1.27 ± 0.18 D postop 1 month after surgery (*p* > 0.05))^20^ when a temporal incision was made. In addition, Garzón et al. also described not statistically nor clinical differences in temporal incision in low astigmatism corneas in SIA^21^. Consequently, for low myopia and astigmatism and based on the previous mentioned clinical studies, the effect of incision can be neglected in the simulations.

Another main factor in ICL implantation is the vault height. As known, different ICL size led to the different post-op ICL position and vault height. The prediction of suitable size ICL and vault always are a challenge as the IOL in the posterior chamber could have different positions as the haptics are not all in the ciliary sulcus. However, these calculations are reliable as several authors have demonstrated indicating that ICL surgery was safe and predictable. In the Meta-analysis and review published by Packer^22^, it was concluded that all currently reported methods of determining the best-fit size of the ICL achieved similarly satisfactory results in terms of vault. Both sulcus-to-sulcus and white-to-white measurement-based sizing methods did not result in clinically meaningful nor statistically significant differences in vault. In addition, the efficacy index (postoperative UCVA divided by preoperative BCVA) and the safety index (ratio of postoperative to preoperative BCVA) were greater than 1.00 in all cases in which it was reported^22^. Moreover, Kamiya et al. described that the changes in vaulting over time there were not significant regarding the refractive error^23^. Based on showed results by these studies, in our simulations we have used an optimized ICL size and vault height for each patient.

Considering the impact of the aforementioned factors, the ICL surgery minimally should modify the cornea and therefore the final quality of vision will be mainly determined by the interaction between the aberrations generated by the cornea and those of the ICL itself (which as we have shown in Table 4, were very small). That is why the MTF and PSF curves and optotypes vision simulation of the eye model and the ICL model are so similar in all eyes. In conclusion, the final quality of vision with ICL implantation will be determined by the pre-surgery optical quality of the cornea and not by the ICL optics itself.

Another finding of this work is that, as several authors has been reported, the spherical aberration and coma increased after LASIK surgery^5,7^. Our results showed this trend both 3 mm and 4.5 mm pupil size. Furthermore, if the pre- and post-operative corneal aberrations up to 6 mm were considered (see Table 5), the spherical aberration and coma clearly increased. These results agree with the bibliography where pupil sizes higher than 5.5 mm were considered^5–7^. Therefore, higher differences between the two surgical techniques are expected as myopia increase.

**Table 5:** Pre-and Post-LASIK surgery Spherical and coma Zernike coefficients of the total cornea measured with the PENTACAM for the four eyes. High Order RMS aberrations (HORMS) were calculated onsidering the Zernike coefficients from the third to eighth orders.﻿

Eye 1	3 mm		4.5 mm		6 mm	
C(n,m)	PRE	POST	PRE	POST	PRE	POST
C(3, − 1)	− 0,005	0,043	− 0,021	0,042	− 0,025	− 0,138
C(3,1)	− 0,006	− 0,045	− 0,039	− 0,13	− 0,141	− 0,194
C(4,0)	− 0,011	− 0,02	0,046	0,008	0,223	0,377
HORMS	**0,018**	**0,073**	**0,074**	**0,17**	**0,28**	**0,511**

Eye 2	3 mm		4.5 mm		6 mm	
C(n,m)	PRE	POST	PRE	POST	PRE	POST
C(3, − 1)	− 0,024	− 0,059	− 0,042	− 0,053	− 0,045	0,207
C(3,1)	− 0,003	− 0,069	− 0,032	− 0,18	− 0,113	− 0,272
C(4,0)	0,009	− 0,02	0,064	0,007	0,247	0,364
HORMS	**0,044**	**0,102**	**0,123**	**0,215**	**0,321**	**0,551**

Eye 3	3 mm		4.5 mm		6 mm	
C(n,m)	PRE	POST	PRE	POST	PRE	POST
C(3, − 1)	− 0,006	− 0,017	− 0,04	0,078	− 0,118	0,066
C(3,1)	0,007	− 0,001	0	− 0,051	− 0,043	− 0,118
C(4,0)	0,015	− 0,004	0,088	0,058	0,295	0,428
HORMS	**0,025**	**0,051**	**0,108**	**0,142**	**0,336**	**0,492**

Eye 4	3 mm		4.5 mm		6 mm	
C(n,m)	PRE	POST	PRE	POST	PRE	POST
C(3, −)	0,049	− 0,038	0,117	0,017	0,197	0,325
C(3,1)	− 0,038	− 0,043	− 0,124	− 0,185	− 0,313	− 0,508
C(4,0)	− 0,003	− 0,013	0,043	0,018	0,265	0,329
HORMS	**0,065**	**0,071**	**0,193**	**0,222**	**0,506**	**0,735**

In this paper has been shown a methodology allows to obtain reliable results in a relative short time, with a computation of the potential quality of vision of the patients before ICL implantation, only requiring preoperative ocular parameters (including topography) and the wavefront pattern of the ICL. Regarding the prediction of the post-LASIK optical quality, it could be also possible if the algorithm of ablation to be applied was known a priori. Furthermore, the PSF analysis and simulation of optotypes vision can also help ophthalmologists to take decisions. However, it is important to know that the simulated vision of optotypes is not considering the neural processing part and consequently the real results are expected to be better.

This study was focused on patients with low myopia and astigmatism and with standard pupil size. It would be interesting in order to generalize these results to consider other possible situations. For example, patients with astigmatism, smaller o larger pupil size or irregular corneas could be conducted. However, in case of astigmatism, the use and characterization of a toric ICL would be required.

Next step could be to apply simple LASIK algorithms and real pre-surgery topographies to be compared with ICL implantation. As these algorithms become more accurate, we will be able to improve the prediction of the results previous any type of surgery.

In conclusion, a new methodology to predict the effects of two different refractive surgery surgical technics is proposed. According to the simulations of the current study, it is necessary to establish optical quality criteria to decide when the implantation of a PIOL would provide better results than LASIK surgery, although our preliminary clinical study indicated that ICL implantation will provide better optical quality of vision if the patient's cornea was not too aberrated.
